# Retrospective analysis of open reduction and internal fixation of coronal plane fractures of the capitellum and trochlea using the anterolateral approach

**DOI:** 10.1051/sicotj/2017063

**Published:** 2018-03-16

**Authors:** Yashwant Singh Tanwar, Yatinder Kharbanda, Atin Jaiswal, Vikas Birla, Ramsagar Pandit

**Affiliations:** 1 Department of Orthopedics, Apollo Hospital, Sarita Vihar, Delhi 110076 India; 2 Maya Hospital, Farrukhabad, Uttar Pradesh India

**Keywords:** Capitellum Fracture, Trochlear fracture, Herbert screw, Anterolateral approach elbow

## Abstract

*Introduction*: Coronal plane distal humeral injuries are relatively rare. Numerous classification systems have been proposed as the complexity of these fractures has been realized. We in the present series of ten patients describe the surgical technique of Open Reduction and Internal Fixation of Coronal plane fractures of the distal humerus with headless compression screws performed using the anterolateral approach.

*Material and method*: It was a retrospective study, the data collected from March 2010 to 2015 was analysed and the final outcome was assessed using the DASH score. Out of a total of 13 patients with distal humerus coronal plane fractures, 10 patients were available for follow up. The X-rays and CT scans were reviewed and the fractures were classified according to Dubberley and Bryan and Morrey classification. Radiographic were evaluated for presence of union or nonunion, avascular necrosis, joint line step-off (none/1-mm/>1-mm), hardware failure and instability.

*Results*: The average age was 41 years. The average DASH score in our study was around 24. The time to union ranged between 8–12 weeks with the average time being around 10 weeks. One patient had post traumatic Arthritis radiologically classified as Broberg and Morrey Type 2 and one patient had Heterotrophic ossification Brooker Grade 1.

*Conclusion*: Open reduction and internal fixation of coronal shear fractures of capitellum and trochlea using headless screw compression via the antero-lateral approach is a reliable treatment modality and results in stable fixation with restoration of a functional arc of motion.

*Level of evidence*: IV

## Introduction

Isolated coronal plane fractures of capitellum and trochlea are rare injuries. Coronal plane fractures of the Capitellum were first described by Cooper and later a more detailed description was given by Hahn, Steinthal and Kocher after whom they have been named. Such injuries usually result from low energy trauma; usually from fall on an outstretched hand with the elbow in extension. As the centre of rotation of Capitellum is anterior to the humeral shaft, it leads to the transmission of a shearing force resulting in a coronal fracture of the distal humerus. Trochlear fracture may result from transmission of a similar force from the coronoid. A higher female preponderance is explained on the basis of a larger carrying angle leading to greater transmission of contact forces to the lateral column [1]. The fracture may occur after an episode of acute elbow dislocation/instability. Capitellum and trochlea may be sheared off by the radial head and coronoid following the reduction of a posterolateral subluxation or dislocation of the elbow. This might also explain distribution of damage from anterior to posterior, from lateral to medial in a sequential manner as described in elbow dislocation, commonly known as Circle of Horii.

Capitellum fractures may also be secondary to recurrent posterolateral instability of the elbow. Due to repeated subluxation and impingement of the radial head against capitellum, an osteochondral fracture of the posterolateral margin of capitellum may occur. It has been termed as Osborne-Cotterill lesion as they were first to describe it [2]. The lesion is similar to Hill-Sachs lesion seen in humerus secondary to recurrent shoulder dislocation.

The most commonly used classification system is that of Bryan and Morrey [3]. Originally there were three types and a fourth type was later added by Mc Kee et al. [4]. Type I also known as Hahn-Steinthal fracture involves a substantial portion of Capitellum bone in the coronal plane. Type II also known as Kocher-Lorenz fracture is an Osteo-chondral fracture involving a shell of the articular cartilage with little bone. Type III − comminuted fracture. Type IV McKee modification − coronal plane fracture involving capitellum and part of trochlea as a single segment.

AO classification [5] although quite comprehensive and detailed, is too complex for daily use. Distal Humerus is anatomically labelled as region 13 and partial articular coronal plane fractures are sub-grouped as type B3. These are then further classified as follows:

- 13B3.1: isolated capitellar fracture;
- 13B3.2: isolated trochlear Fracture;
- 13B3.3: combined capitellum and trochlea fracture.

**Figure 1:**
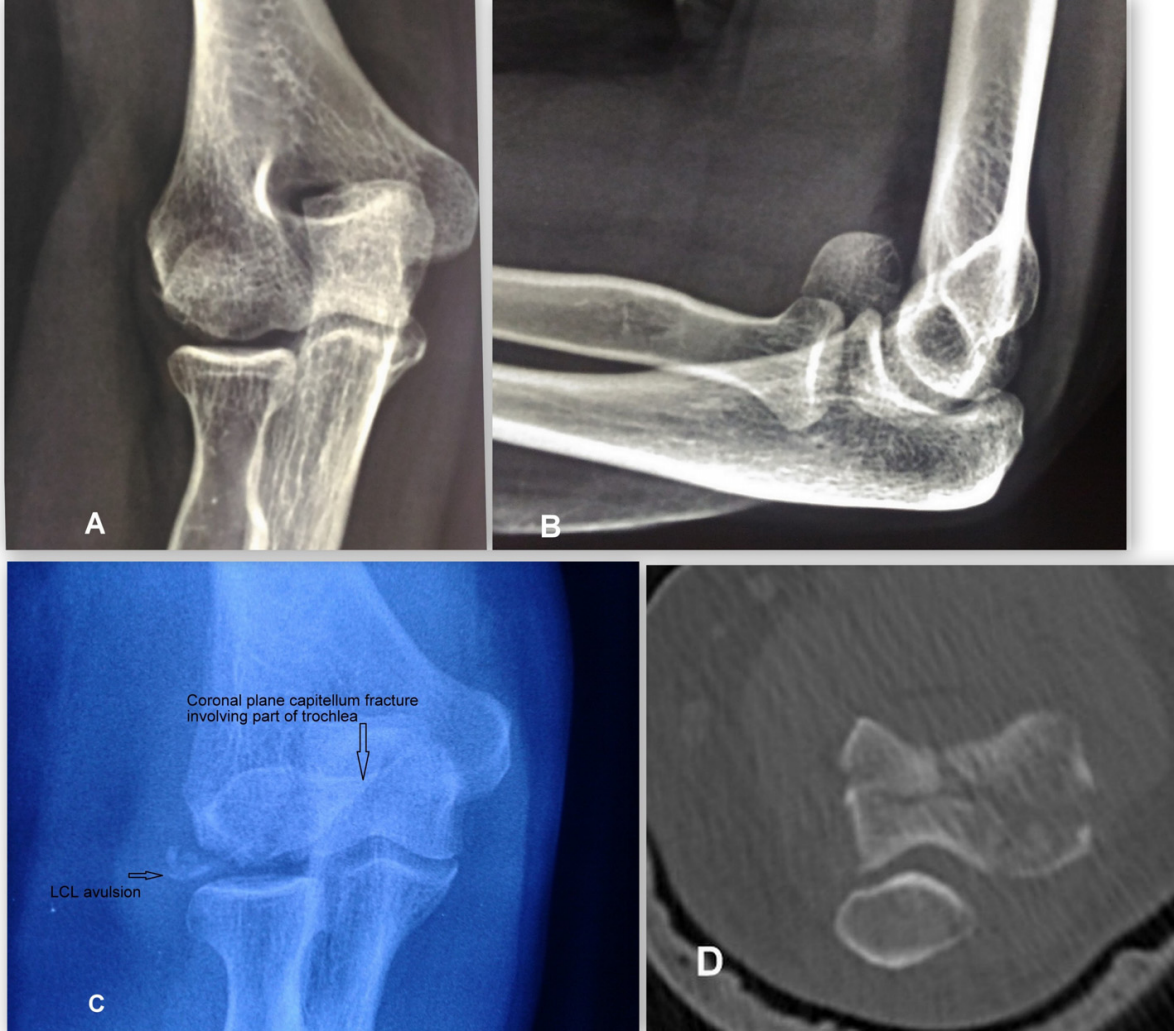
The different Dubberley fracture types. A and B − Type 1 fracture. C − Type 2 fracture. D − Type 3B fracture.

Dubberley et al. [6] proposed a classification system that was correlated to the clinical outcome. Type-1 injuries involved primarily the capitellum with or without the lateral trochlear ridge (Figures 1a and b); Type-2 injuries involved the capitellum and trochlea as one piece (Figure 1c); Type-3 injuries consisted of fractures of both the capitellum and trochlea as separate fragments (Figure 1d). This classification further sub classifies the fractures as A or B based on the presence or absence of postero lateral comminution.

**Figure 2:**
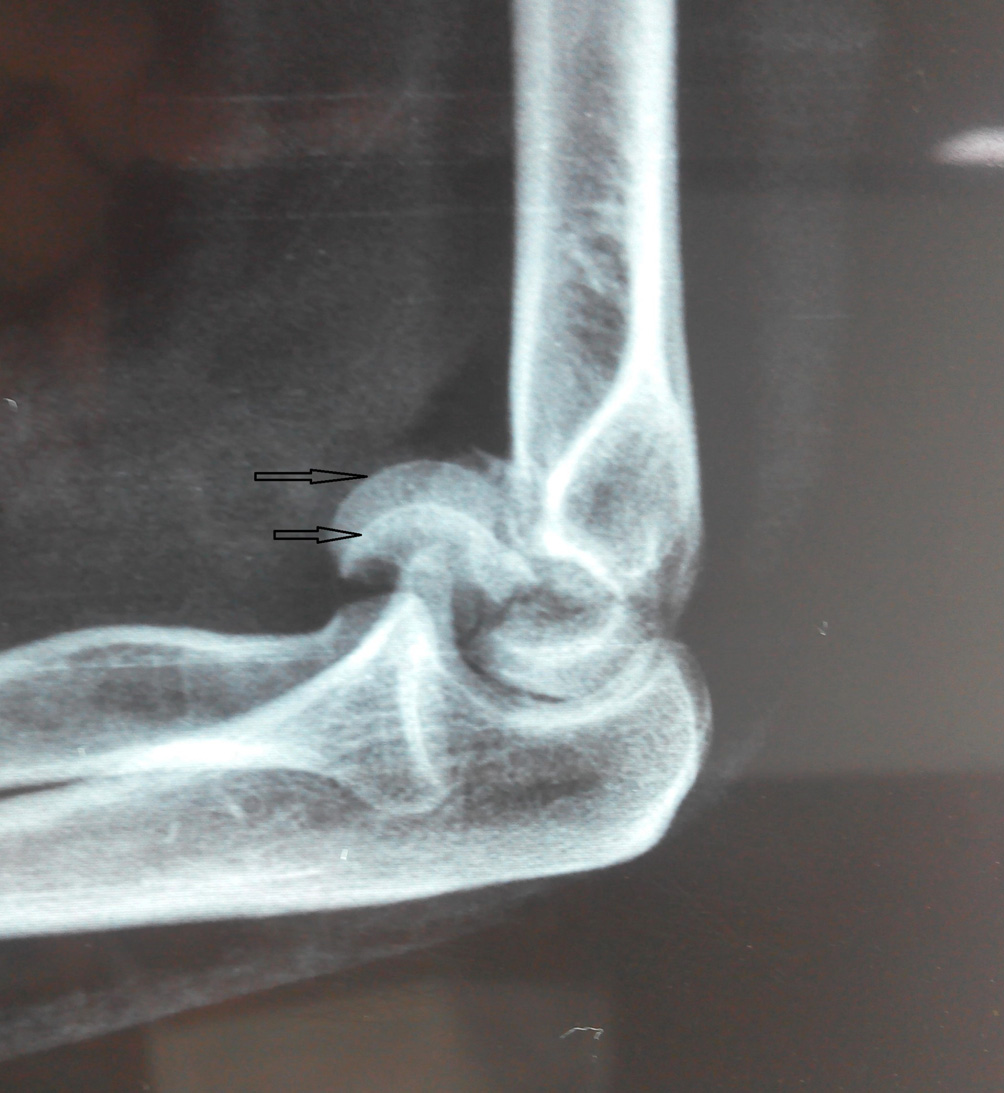
Classical double arc sign signifying involvement of trochlea in the coronal fracture.

**Figure 3:**
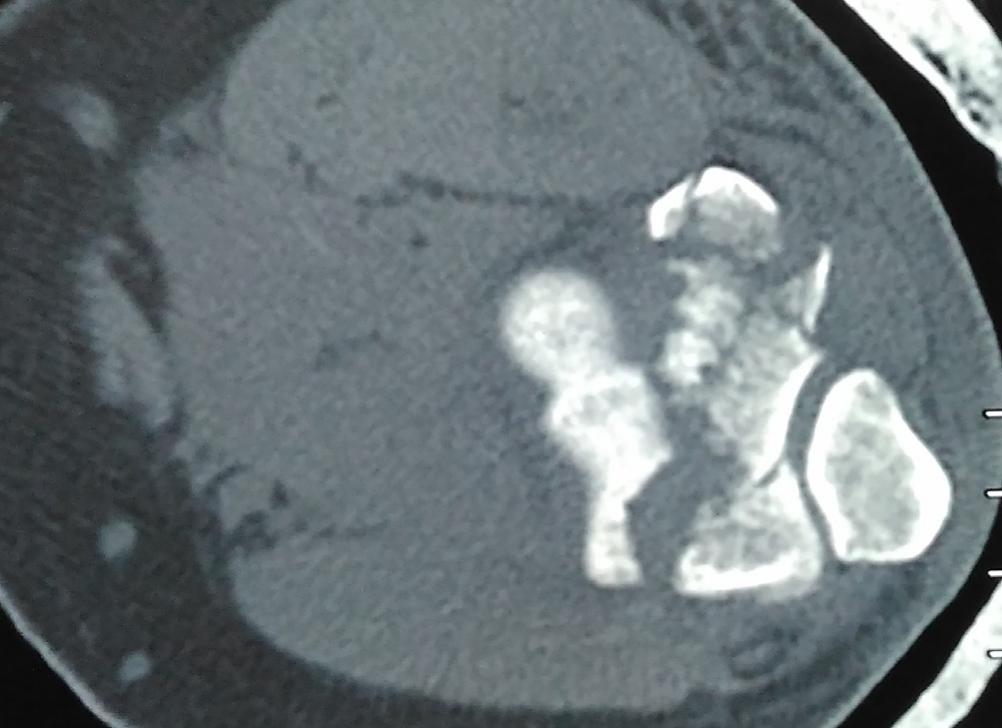
Axial Section CT scan showing a complex coronal plane fracture of distal humerus with posterior and lateral comminution.

Patients usually present with pain and swelling around the elbow region. Examination of Bryan and Morrey type I fractures shows a mechanical block in elbow flexion, which is due to the anteriorly displaced fragment, whereas type II fractures usually show a mechanical block in extension as a result of the posteriorly displaced osteo-chondral fragment. Pain over the medial aspect may indicate an underlying MCL avulsion or tear. Lateral tenderness may indicate an associated radial head fracture or LCL injury (Figure 1c). X-rays especially lateral view are helpful in making the diagnosis. The typical semilunar fragment is displaced anteriorly and superiorly. In case of type 4 fractures in which the fragment consists of capitellum and the lateral half of trochlea, “Mckee's double arc sign” is seen (Figure 2). The two arcs are due to the subchondral bone of capitellum and trochlea. CT scan provides a much more detailed view and should be performed in the all the cases to know about the displacement pattern, amount of comminution, involvement of posterior cortex, epicondyles and radial head. The fracture pattern is usually more complex than that is evident on simple X-rays (Figure 3).

There are many treatment options described in the literature ranging from conservative management to surgical fixation with headless screws or K-wires. We in the present series of ten patients describe the surgical technique of ORIF of Coronal plane fractures of the distal humerus with headless compression screws performed using the anterolateral approach at a single centre by the same senior surgeon (YK). It was a retrospective study, the data collected from March 2010 to 2015 was analysed and the final outcome was assessed using the DASH score.

## Material and methods

Out of a total of 13 patients with distal humerus coronal plane fractures, 10 patients were available for follow up. Out of these 10 patients there were 3 males and 7 females. The average age was 41 years. Four patients sustained the injury due to Road traffic accident whereas the rest 6 had fall from height. Time interval between injury and surgery ranged from 1 to 5 days, with most of the patients (6) having undergone the surgery within 24 hours. Inclusion criteria were: Age between 18 to 60 years; patients having coronal plane distal humerus fracture, patients who presented within 10 days of injury having no distal neurovascular deficit; patients having no other bony injury in the ipsilateral upper limb. Patients having pre-existing deformities of ipsilateral shoulder, elbow or hand and patients in whom anterolateral approach was not used were excluded from the study. This approach was not universally used in all coronal plane distal humerus fractures rather the choice of surgical approach was guided by numerous variables as discussed later (Table 2). All the surgeries were performed by the same senior author (YK) at one institution only.

**Table 2:**
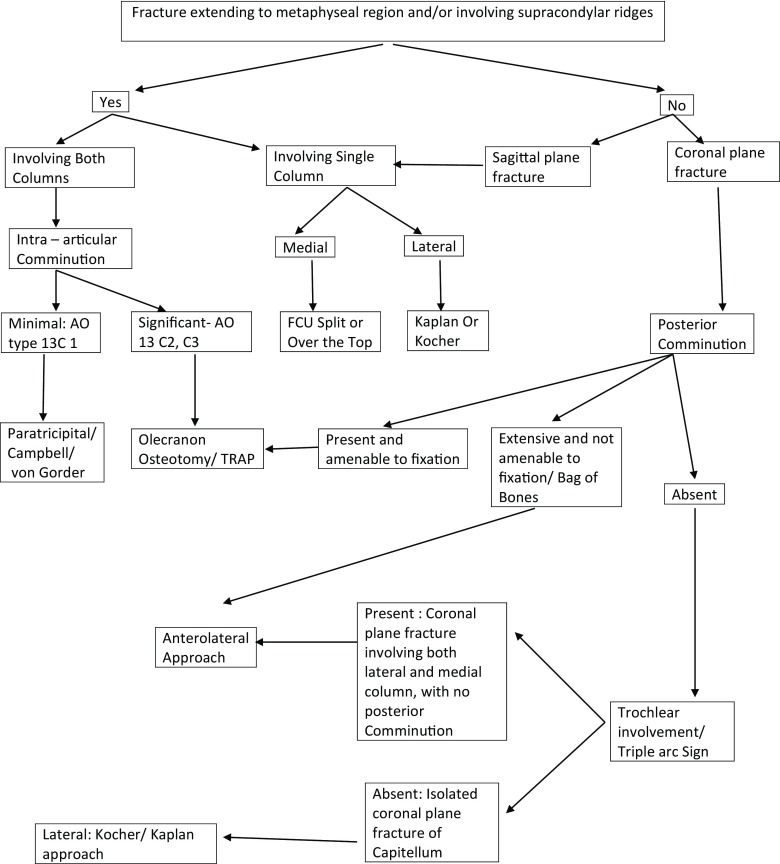
Algorithm for guiding the selection of appropriate approach in Distal Humerus Fracture.

### Surgical technique

**Figure 4:**
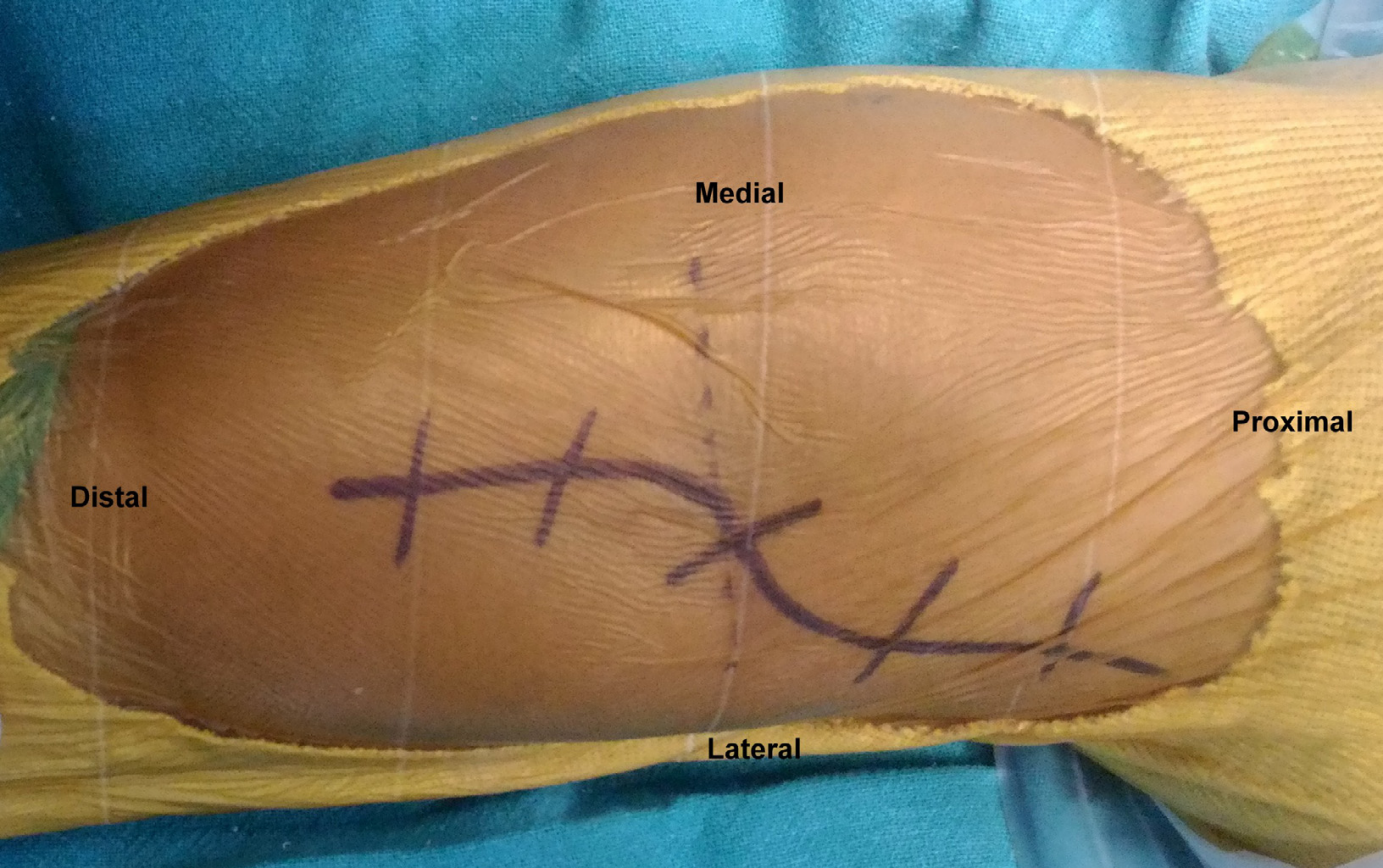
The curved S shaped skin incision.

**Figure 5:**
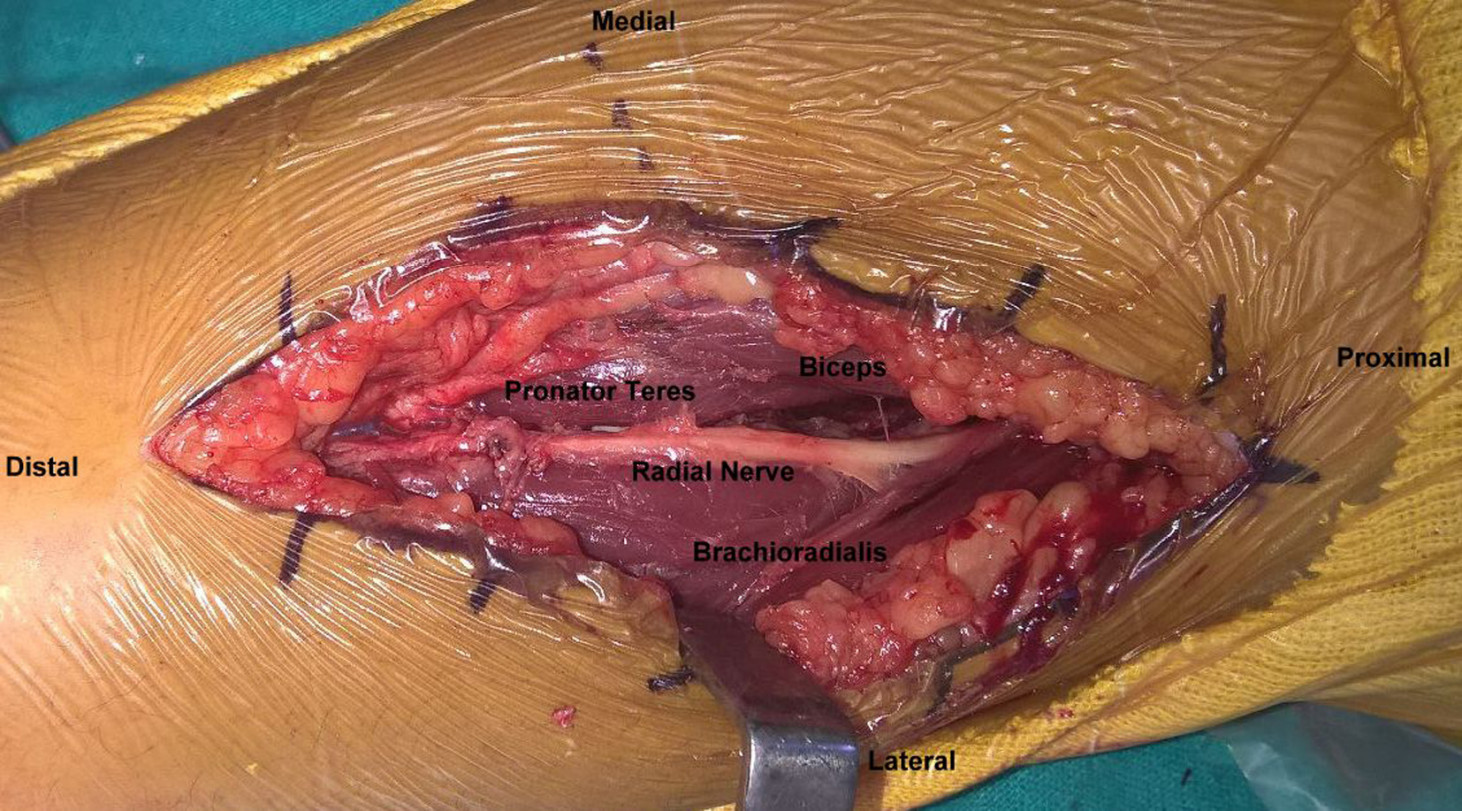
The deep dissection planes and the isolation of radial nerve.

**Figure 6:**
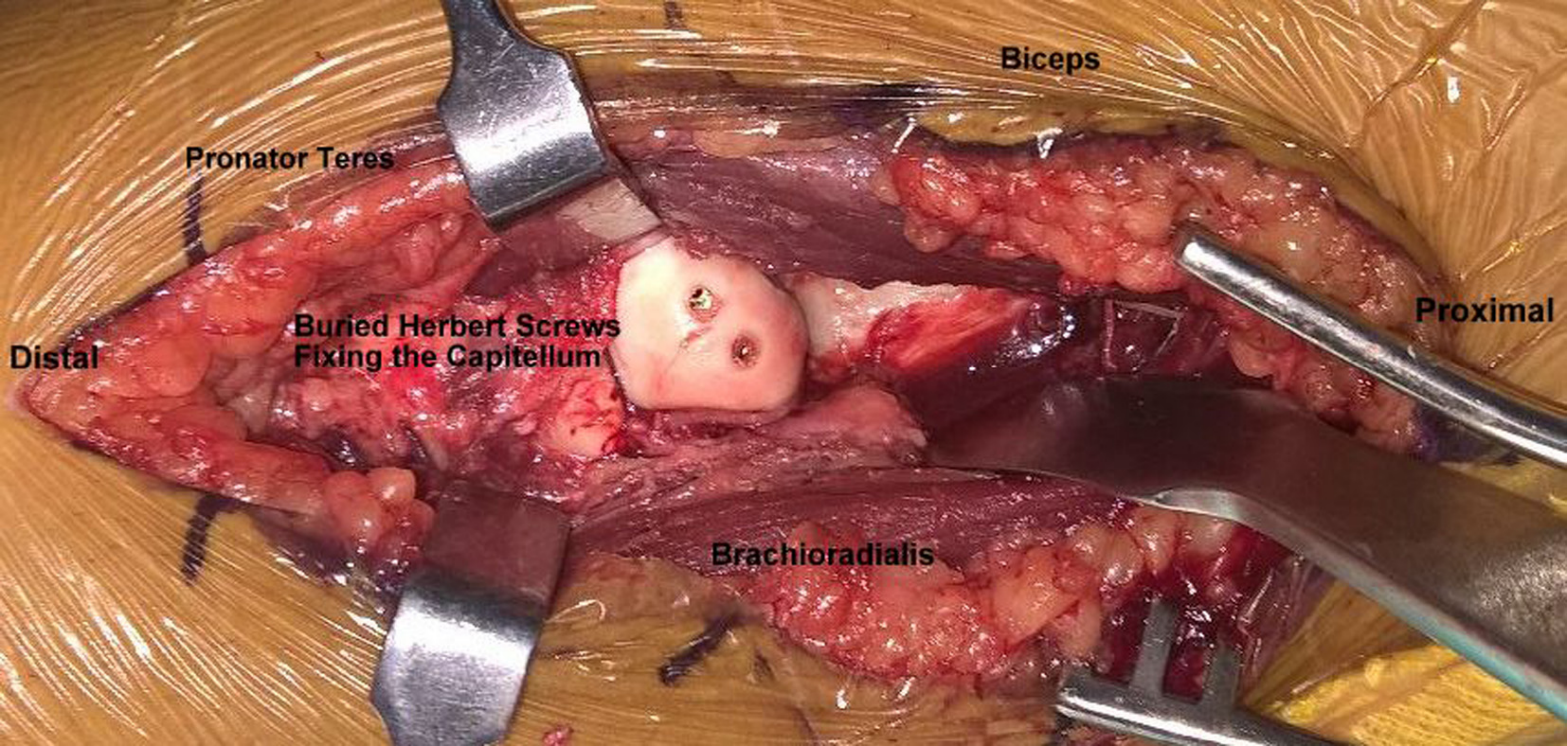
Intra operative image of fixation using buried Herbert screws.

**Figure 7:**
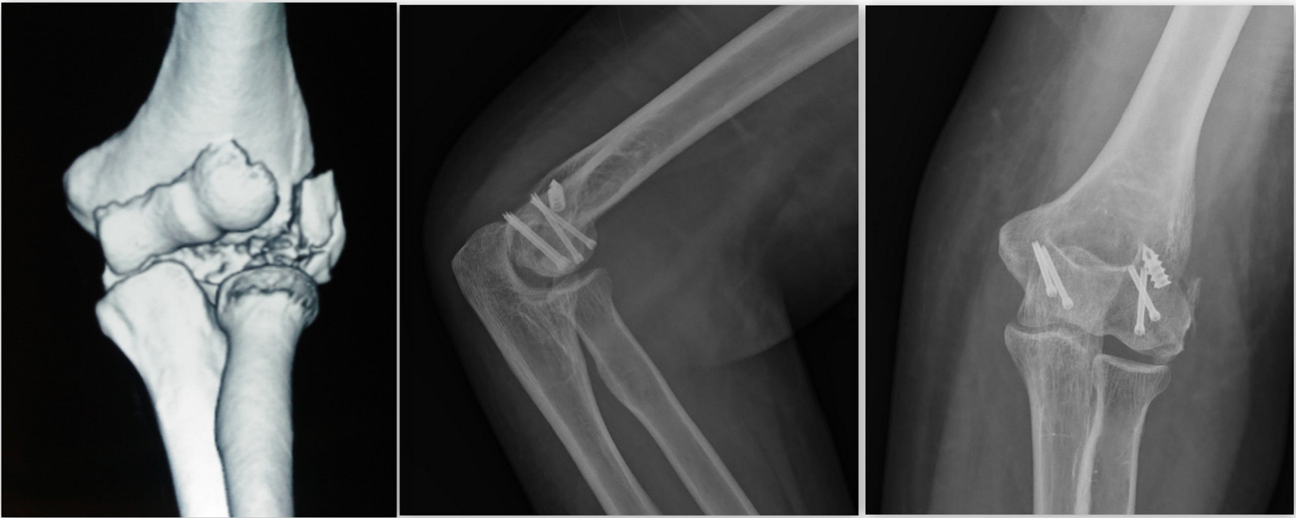
LCL avulsion fracture and fixation of LCL with suture anchor.

The patient is placed supine and a tourniquet is placed on the upper arm. We in the present series used an Anterolateral approach in all the cases. The arm is exsanguinated using an Esmarch bandage and tourniquet is inflated. A curved S shaped incision is made, with the superior limb being along the lateral border of biceps (Figure 4). The incision is then curved medially at the level of elbow joint to avoid crossing a flexion crease at 90 degrees and distal limb of the incision is curved along the medial border of Brachioradialis. The lateral cutaneous nerve of the forearm is identified and preserved as it becomes superficial lateral to the biceps tendon. Proximally the plane between Brachialis and Brachioradialis is developed and distally the plane between medial border of Brachioradialis and lateral border of Pronator teres is developed (Figure 5). The radial nerve is identified proximally at the level of elbow joint between Brachialis and Brachioradialis (Figure 5). The nerve is traced distally and its three branches namely: Superficial Radial nerve which continues distally below the Brachioradialis, Posterior Interosseous which pierces the Supinator and the branch to extensor carpi radialis brevis (ECRB) which enters the muscle almost immediately, are identified and preserved throughout the procedure. Small branches of the recurrent radial arterial arcade are identified and ligated so that the Brachioradialis can be mobilized. The elbow joint capsule is incised longitudinally if not already torn and the fracture fragments are identified. Anterior approach provides direct access to anterior aspect of the medial and lateral column. K wires are introduced in the fracture fragments, used as joystick to manipulate them into anatomical reduction, and then driven across the fracture site into opposite cortex. Definitive fixation is achieved with the help of headless compression cannulated 2.4 mm Herbert screws, placed at right angles to the fracture site (Figure 6). While exposing the LCL avulsion, elbow should be pronated and a varus stress should be placed, so that the lateral epicondyle can be visualized adequately. Repair of the avulsed LCL fragment is therefore done last, so as avoid supinating the forearm after wards. Associated injury of the LCL is repaired by using a running interlocking suture, which is fixed to its anatomical origin from the lateral epicondyle or a Suture Anchor placed in the Lateral Epicondyle bed. It's not necessary to fix the avulsed fragment into exact centre of rotation of humeral capitellum, as is done while reconstructing LCL. Rather the avulsed fragment should be fixed at its anatomic position as judged intra-operatively (Figure 7).

Post-operatively arm is placed in a Soft Dressing and arm sling for two weeks. Gentle active and active assisted range of motion exercises are started thereafter. During the weeks 2–4, stress was placed on achieving adequate range of motion: Flexion/Extension and Pronation/Supination. If concomitant LCL repair was done, then elbow was kept pronated for 4 weeks. After 4 weeks Muscle strengthening exercises were started, however arm pouch sling was continued till 6 weeks. Physical therapy sessions were continued for 8–10 weeks. A hinged Elbow Brace was used in two patients for 4 weeks in whom it was felt intra-operatively that fracture fixation was not stable enough due to extensive comminution and an associated LCL injury. Final assessment was done with DASH score at follow up.

## Results

**Figure 8:**
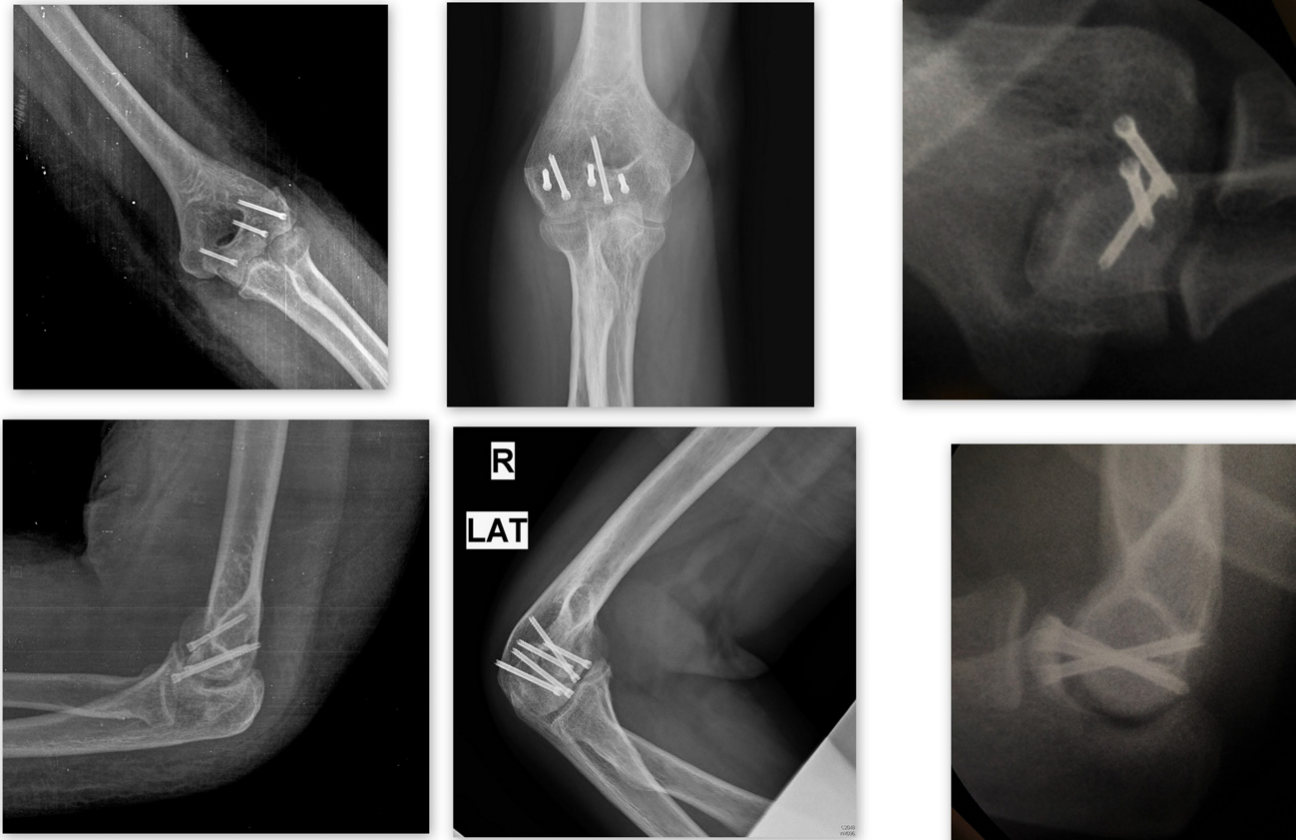
Post op X-rays showing union of fracture.

The data was analysed from March 2011 to 2015, out of a total of 13 patients with distal humerus coronal plane fractures, 10 patients were available for follow up. The X-rays and CT scans were reviewed and the fractures were classified according to Dubberley and Bryan and Morrey classification by two authors (YK, YST). Radiographic were evaluated for presence of union or nonunion, avascular necrosis, joint line step-off (none/1-mm/>1-mm), hardware failure and instability (Figure 8). Associated LCL injury was repaired in two patients using suture anchors. Arthrosis was evaluated subjectively using the system described by Broberg and Morrey [7] as: grade 0 if there was no signs of arthritis; grade 1 if there was slight joint-space narrowing and minimal osteophyte formation; grade 2 if there was moderate joint-space narrowing and osteophyte formation, or grade 3 if there was severe joint-space narrowing with gross destruction. Heterotopic ossification (HO) was classified using the Brooker classification [8] applied to the elbow: class I was defined as islands of bone within the soft tissues; class II required the presence of ectopic bone from the humerus, radius, or ulna, leaving at least 1 cm between opposing surfaces; class III required ectopic bone from the humerus, radius, or ulna, reducing the space between opposing bone surfaces to less than 1 cm; and, lastly, class IV HO demonstrated apparent bone ankylosis of the elbow joint. Four fractures were sustained as a result of Road traffic accident whereas the rest were due to falls. DASH (Disabilities of the Arm, Shoulder and Hand) score was evaluated. The DASH Outcome Measure is a 30-item, self-report questionnaire designed to measure physical function and symptoms in people with any of several musculoskeletal disorders of the upper limb. The normal DASH score in the general population has been reported to be around 10 with a standard deviation of 14.68 [9]. The average DASH score in our study was around 24. The time to union ranged between 8–12 weeks with the average time being around 10 weeks (Table 1). We did not encounter any case with AVN or non-union. One patient had post traumatic Arthritis radiologically classified as Broberg and Morrey type 2 which was managed conservatively. One patient had Heterotrophic ossification Brooker Grade 1, which was asymptomatic. The mean time between injury and surgical intervention was 2 days (range 1–5 days).

**Table 1:** Depicting the various observations made in the study.

Sr. No.	Dubberley type	Bryan and Morrey type	Mechanism of injury	Interval between injury and surgery	Associated injuries	Posterior Cortex involvement	Sex	Age	ROM	DASH Score	Time to union in weeks	Complication
1	2 A	1	RTA	1	LCL	–	M	28	15–120	27.5	10	–
2	2 A	4	RTA	3	–	–	M	32	10–130	18.3	10	–
3	1 A	1	Fall	1	–	–	F	41	5–135	15	8	–
4	3 B	3	Fall	5	–	Present	F	49	20–110	35.8	12	Heterotrophic Ossification Grade 1
5	2 B	3	Fall	1	LCL	Present	F	38	10–120	29.2	10	Grade 1 Arthritis
6	1 A	1	RTA	1	–	–	F	52	5–130	14.2	8	–
7	2 B	3	Fall	4	–	Present	F	55	15–130	25.8	12	–
8	2 A	4	Fall	1	–	–	F	46	10–135	21.7	10	–
9	2 A	4	RTA	1	–	–	M	39	5–130	16.7	8	–
10	3 A	4	Fall	2	–	–	F	37	10–125	30	12	–

## Discussion

Isolated coronal plane articular fractures are uncommon and challenging fractures which are classically described in the distal femur and distal humerus. Their rarity and complexity along with the challenges in approaching and fixing them with unfamiliar approaches, prompted us to analyse and report our experience of surgical fixation using the anterolateral approach to elbow.

**Figure 9:**
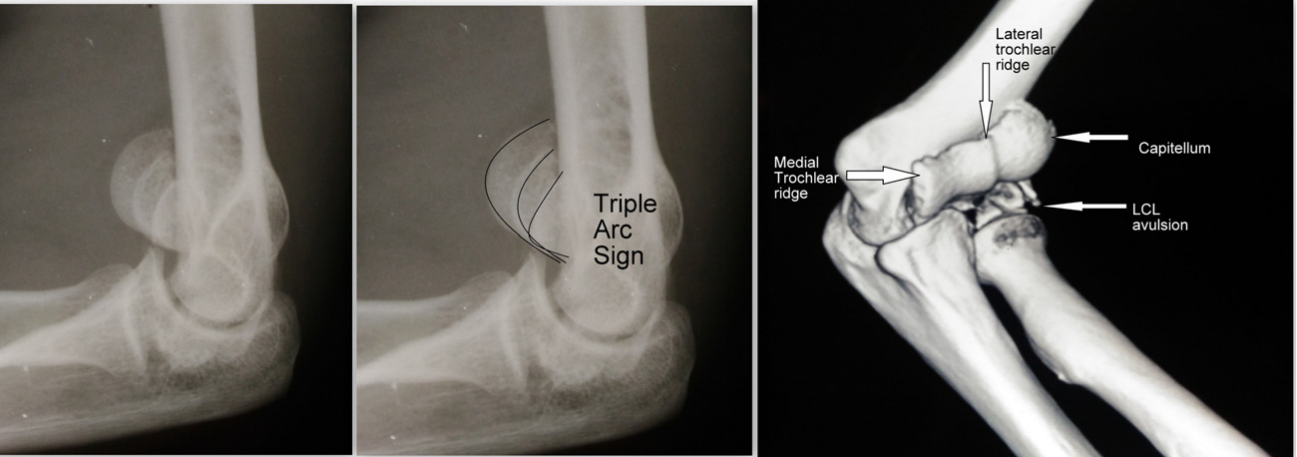
X-rays showing Triple Arc Sign.

Coronal plane injuries can be easily missed on the radiographs, especially only if AP views have been taken. Greenspan and Norman described a modified lateral view of the elbow in which the radiographic plate is placed under the elbow with the arm abducted 90° at shoulder and elbow flexed to 90°. Radiographic beam is centered on the radial head, and angled 45° dorsoventrally, eliminating the overlap of the humeroulnar and humeroradial articulations thus outlining the radial head and capitellum in profile [10]. McKnee described the double arc sign indicating that a segment of trochlea is involved along with Capitellum. We came across “Triple Arc sign” in a patient in whom the whole of trochlea along with Capitellum was fractured in Coronal plane (Figure 9). One arc represented the Capitellum and the other two represented medial and lateral trochlear ridges.

A high index of suspicion is required and if in doubt a CT should always be performed. CT also give additional information regarding any associated occult fractures (such as radial head) and associated ligamentous injuries. Ring observed that the complexity so called “isolated” capitellar fractures was underestimated on plain X-rays. He described five different anatomic zones and classified the fracture patterns according to them: capitellum and the lateral aspect of the trochlea; lateral epicondyle; posterior aspect of the lateral column; posterior aspect of the trochlea; medial epicondyle. He also coined the term Apparent capitellar fractures as it emphasizes the “need to look more closely” [11].

**Figure 10:**
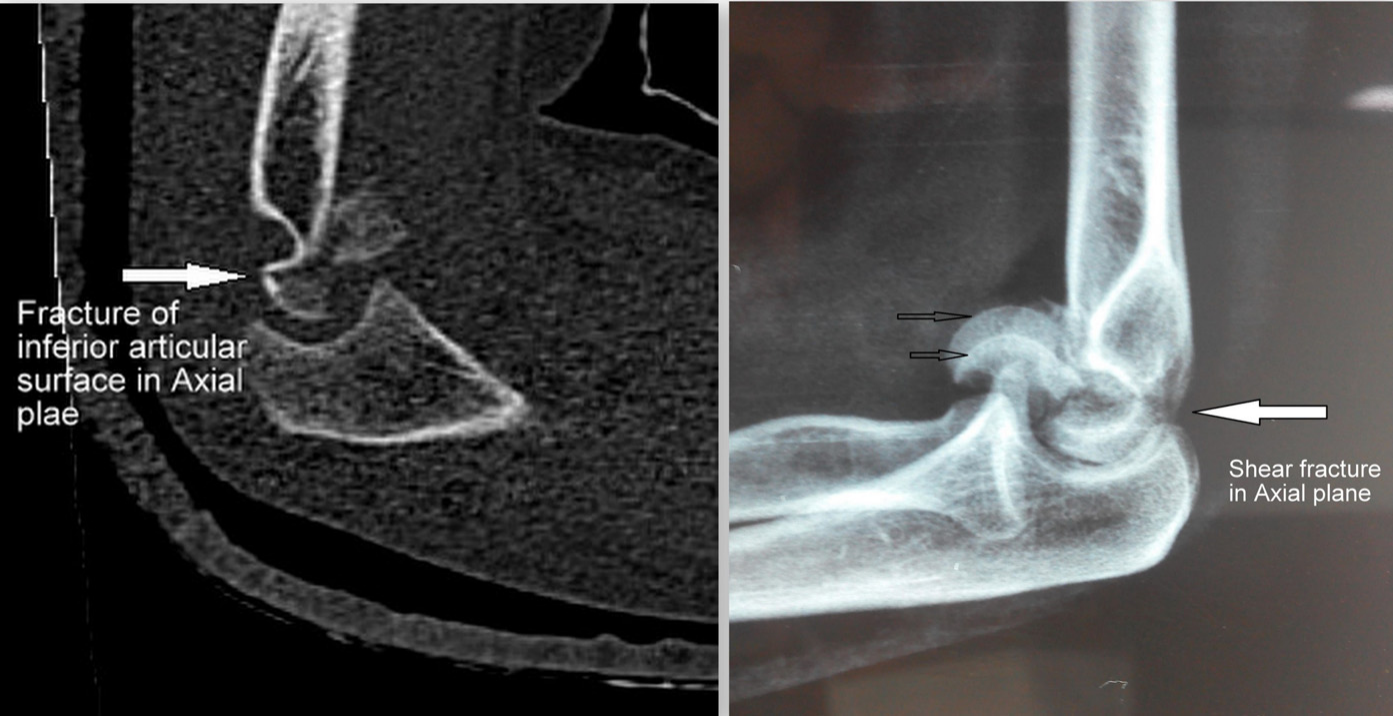
Sagittal section CT scan and X-ray images showing fracture of the inferior articular surface in axial plane.

There are numerous classification systems. We in the present series classified the fractures according to Bryan and Morrey and Dubberley classification systems. An important aspect of classification of fracture is that it gives an idea about the severity of the injury and the prognosis. All patients with posterior cortex involvement (Dubberley type B) had high DASH scores (35.8, 29.2 and 25.8) indicating poor results. We observed that in a couple of patients there was a fracture of the inferior articular surface which was separate from the anterior and posterior segments (Figure 10). Such a separate fracture has not been subclassfied separately in any of the classification system. However Ring did mention this in his classification of five fracture types: Type 1 – a single articular fragment that includes the capitellum and the lateral portion of the trochlea. Type 2 – Type-1 fracture with an associated fracture of the lateral epicondyle. Type 3 – Type-2 fracture with impaction of the metaphyseal bone behind the capitellum in the distal and posterior aspect of the lateral column. Type 4 – Type-3 fracture with a fracture of the posterior aspect of the trochlea. Type 5 – Type-4 fracture with fracture of the medial epicondyle [11]. We did not feel the need to fix this segment separately as in both the cases the segment was well contained within the trochlear notch of ulna. We also concluded that owing to the extremely complex nature of these injuries, there are still many fracture patterns which cannot be clearly classified into one category.

Treatment options include: Closed Reduction and immobilization in Cast [12], fragment excision, open reduction and internal fixation, arthroscopic assessment and fixation and hemiarthroplasty [13] or Total Elbow Arthroplasty.

ORIF has been shown to provide the most reliable and predictable return to function. The approach to ORIF and the means of fixation have varied diversely [4,14,15]. The approach to fixation can be anterolateral [16], Lateral, [17,18] posterior [11,17] or rarely combined medial and lateral [15].

The lateral approach either utilizes the interval between extensor digitorum communis and ECRB as described by Kaplan (18) or the Kocher interval between Anconeus and ECU Extensor Carpi Ulnaris [19]. For better exposure the origin of the wrist extensors and lateral collateral ligament (LCL) may need to be elevated from the lateral epicondyle. LCL elevation can potentially disrupt the blood supply to Capitellum and exposure and visualization of the medial trochlear fragment and posterior comminuted area is difficult [14].

Posterior exposure can be either via Chevron Olecranon Osteotomy approach or the Triceps reflecting Bryan and Morrey approach. Advantages of posterior approach include direct visualization of the posterior comminuted area (Table 2), placement of screws in the non-articular surface and application of a stabilising plate on the posterior column. Besides the inherent shortcomings of a palpable implant and the risk of non-union at the osteotomy site, the posterior approach has two more potential drawbacks; firstly if an intra-operative decision to perform a total elbow arthroplasty has been taken, then an olecranon osteotomy precludes such a procedure; secondly it damages the already precarious blood supply to Capitellum.

In the present series anterolateral approach was used in all the cases. To the best of our knowledge there has been only one case series describing the use of this approach [16]. The main advantage of using an anterior approach is the adequate exposure of the medial column, which may be required if there is extension of the fracture plane medially into the trochlea as in Bryan and Morrey type 4 [20]. One of the drawbacks of Anterolateral approach is inadequate posterior exposure to address the posterior comminution. CT scans should be carefully evaluated regarding the extent of comminution and whether it is amenable to surgical fixation. In cases with extensive posterior comminution, it is best to approach the fracture anteriorly where sufficient bone is available for fixation of large displaced fragments and address the posterior comminution anteriorly. After removing the anterior fragments, varus stress is given along with traction to open/sublux the joint. The posterior fragments can now be addressed by manipulating/pushing the fragments back in place. Any metaphyseal void thus crated can be filled with graft. If however the posterior comminution is deemed feasible for surgical fixation then a posterior approach should be considered either in isolation or in addition to anterolateral approach (Table 2). Apart from this a simultaneous posterior exposure may also be required if a decision is taken to proceed to total Elbow Arthroplasty.

The major nutrient artery of the humerus terminates proximal to the distal humerus so the elbow appears to be relatively dependent on local osseous perforating vessels for its blood supply. There are three arterial arcades (lateral, medial, and posterior) around the elbow. The radial and medial collateral arteries (branches of the profunda brachii) anastomose with the ascending inter-osseous and radial recurrent arteries to form the lateral arcade, which supplies the capitellum from its posterior aspect. The intraosseous pathway of the vessels shows a hypovascular area in the trochlear groove, making this area theoretically more susceptible to nonunion or avascular necrosis [21]. Exposure via the Anterolateral approach avoids the posterior soft tissue dissection and thus the main blood supply to the capitellum is preserved, theoretically decreasing the chance of AVN.

The fixation modalities can be Herbert screws, [16] Bio-absorbable screws/pins [22], hinged external fixator [23], Accutrac Screws [18], K wires [19], partially threaded Cortical or Cancellous Screws [17], or anti-glide plate. K wires alone or in combination can be used when the fragments are small like in Bryan and Morrey Type 2 injuries. However there is risk of implant migration and loosening. Given the close proximity of neurovascular structures, K wire placement seems to be dangerous. Elkowitz et al showed that the headless screws provided more stable fixation of capitellum fractures in the cadaveric specimens than four-millimeter partially threaded cancellous lag screws and may do so in the clinical setting [24].

We in the present series used 2.4 mm Herbert screws for all the cases. We did not encounter any fragmentation of the anterior part while inserting the screws. In most of the cases (8 out of 10) the fixation was judged to be stable enough to allow early range of Motion exercises, without additional support. In most of the cases accurate reduction is possible and when there are more than one fragments, careful attention should be given to the orientation and position of each one. Sometimes however the fracture fragments don't fit into the fracture bed which usually happens when there is posterior impaction of the lateral column. In such cases the fracture bed needs to dis-impacted gently and fixed with a Herbert screw. The need for an additional posterior fixation should be judged on table, keeping in mind the benefits offered by improved stability versus the drawbacks of increased soft tissue dissection. We did not find the need to go for supplemental posterior fixation in any of our patients. The lateral epicondyle fragment is rarely large enough to accept a screw or require a plating. We in present series encountered two patients with concomitant LCL injuries both of which were repaired using pull out suture/suture anchor into the anatomical LCL location.

Complications after ORIF include AVN of the fragment, Elbow stiffness, heterotrophic ossification, Post-traumatic arthritis, non-union and Malunion. We did not encounter any case with AVN or non-union, probably owing to a decreased soft tissue dissection and iatrogenic interference with the posterior vascular supply. One patient had post traumatic Arthritis radiologically classified as Broberg and Morrey Type 2 which was managed conservatively. There are many potential drawbacks of the study namely a small sample size, retrospective study design and lack of biomechanical data to assess the strength of fixation.

## Conclusion

Open reduction and internal fixation of coronal shear fractures of capitellum and trochlea using headless screw compression via the antero-lateral approach is a reliable treatment modality and results in stable fixation with restoration of a functional arc of motion. Statistical analysis of our study population demonstrated that the majority of capitellum fractures occur in female patients and involve the nondominant extremity. The drawbacks of this study include a small sample size and a retrospective study design.

The study was approved by the Institutional Review Board. All patients gave the informed consent prior being included into the study. And the study was in accordance with the 1964 Helsinki declaration and its later amendments.

## Conflict of Interest

The authors declare that they have no conflict of interest.

## Funding

There is no funding source.

## Ethical approval

This article does not contain any studies with human participants or animals performed by any of the authors.
